# RNA sequencing data from osteochondroprogenitor populations in synovial joints of mice during murine model of rheumatoid arthritis

**DOI:** 10.1016/j.dib.2020.106570

**Published:** 2020-11-23

**Authors:** Nina Lukač, Vedran Katavić, Alan Šućur, Maša Filipović, Danka Grčević, Nataša Kovačić

**Affiliations:** a Laboratory for Molecular Immunology, University of Zagreb School of Medicine, Zagreb, Croatia; b Department of Anatomy, University of Zagreb School of Medicine, Zagreb, Croatia; c Department of Physiology and Immunology, University of Zagreb School of Medicine, Zagreb, Croatia

**Keywords:** RNA sequencing, Antigen-induced arthritis, Rheumatoid arthritis, Osteochondroprogenitors

## Abstract

The aim of this study was to analyze the transcriptome of TER119^–^CD31^–^CD45^–^CD51^+^CD200^+^CD105^–^ population (further, CD200^+^), potential early osteocondroprogenitors, whose frequency is reduced in the joints of mice with antigen-induced arthritis (AIA) [1]. A population defined by similar surface markers has been previously identified as murine skeletal stem cells in bone [2]. In order to confirm their identity this population was compared to TER119^–^CD31^–^CD45^–^CD51^+^CD200^–^CD105^+^ (further, CD105^+^) cells, which possibly represent committed progenitors, or other non-progenitor population such as synovial fibroblasts. In order to asses changes in CD200+ population in inflammatory setting, it was also compared to the same population from healthy mice. AIA was induced by immunization of mice with methylated bovine serum albumin (mBSA) and subsequent intra-articular injection of mBSA, while non-immunized mice were injected with phosphate-buffered saline at all timepoints. Ten days after intra-articular injection, knee joints were harvested and synovial cells were released by collagenase digestion. Using fluorescence-activated cell sorting, 200–500 cells from selected populations were sorted directly into cell lysis buffer, RNA was reversely transcribed, and first strand cDNA product was amplified. cDNA amplicons were used for library preparation. Bioinformatics analysis was performed using cutadapt [3], HISAT2 [4], Samtools [5] and StringTie [6] tools, and egdeR [7], limma [8], and ClusterProfiler [9] Bioconductor packages. In addition to access to raw data at the NCBI Gene Expression Omnibus repository, this article also provides sample similarity analysis, tables of differentially expressed genes, graphic visualisations of differential expression and gene set enrichment analysis performed on publicly available GO terms. Interpretation of osteochondroprogenitor phenotype of CD200^+^ population based on analysis of presented data is provided in the article “What do we know about bone morphogenetic proteins and osteochondroprogenitors in inflammatory conditions?” [10]. Reuse of this data may help researchers elucidate alterations of synovial stromal and osteochondroprogenitor populations in inflammatory settings and define their role in structural damage in rheumatoid arthritis.

## Specification Table

**Table:** 

Subject	Immunology, Allergology and Rheumatology
Specific subject area	Phenotype of osteochondroprogenitor populations in the synovial joints during murine model of rheumatoid arthritis
Type of data	FASTQ files Tables Figures
How data were acquired	RNA sequencing – NextSeq 500 instrument (Illumina, San Diego, CA, USA)
Data format	Raw (FASTQ files at GSE148130) Processed (raw count values (GSE148130_gene_count_matrix84.csv.gz) and normalized and filtered count values (GSE148130_gene_count_matrix84_normalized_filtered.csv.gz) at GSE148130) Analyzed (with the article and at Mendeley Data [11,12])
Parameters for data collection	AIA was induced in C57BL6 mice as previously described [1]. CD200^+^ cells from joints of non-immunized mice and mice with AIA, and CD105^+^ from mice with AIA were sorted into cell lysis buffer and cDNA amplicons were created using SmartSeq v4 Ultra® Low Input RNA Kit for Sequencing (TakaRa, Kyoto, Japan). Libraries were prepared using Nextera XT DNA Library Preparation Kit (Illumina).
Description of data collection	Libraries were sequenced with a NextSeq 500 instrument (Illumina). Illumina and SmartSeq adapter sequences were removed with cutadapt [3]. Sequences were aligned by HISAT2 [4] and Samtools [5] was used to convert SAM files to indexed, sorted, and merged BAM files. Transcripts were assembled and quantified using StringTie [6]. Count matrices were normalized in egdeR package [7] from Bioconductor. Differences in gene expression were assessed by the *limma voom* algorithm [13] from the limma package [8]. Gene set enrichment analysis was conducted by the ClusterProfiler package [9].
Data source location	Laboratory for Molecular Immunology, Croatian Institute for Brain Research and Department of Anatomy, University of Zagreb School of Medicine Zagreb Croatia
Data accessibility	Raw and processed RNA sequencing data are available at the NCBI GEO repository at GSE148130 (https://www.ncbi.nlm.nih.gov/geo/query/acc.cgi?acc=GSE148130). Sample similarity analysis, differential expression analysis, GSEA are available with the article. Tables with complete results of differential expression analysis (https://data.mendeley.com/datasets/5smkbb8twt/1) and GSEA (https://data.mendeley.com/datasets/432zctddfh/1) are available at Mendeley Data [11,12].

## Value of the Data

• These data present transcriptome analysis of potential osteochondroprogenitor populations which have not yet been assessed in the joints of mice with arthritis and enable comparisons of two different populations, CD200+ and CD105+ cells, present in the inflamed joints as well as comparisons of potential earliest progenitors, CD200+ cells, in healthy and arthritic settings.
• Researchers interested in the gene expression of osteochondroprogenitor populations and alteration of their gene expression in inflammatory setting may benefit from these data.
• These data provide information of the transcriptome of very rare populations, and may enhance further elucidation of the osteochondroprogenitor hierarchy, their expression markers, and phenotype in murine joints.

## Data Description

**Fig. 1:**
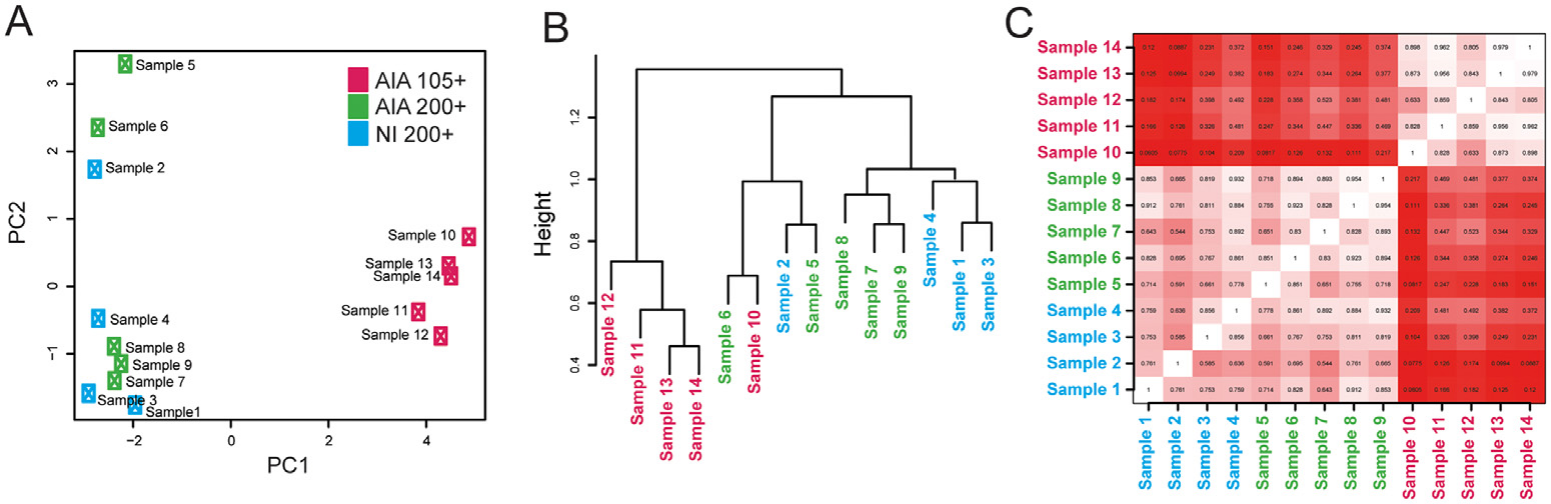
**Sample similarity analysis**. Sample similarity analyses of sorted synovial TER119^–^CD31^–^CD45^–^CD51^+^CD200^+^CD105^–^ cells from non-immunized (NI) mice (NI 200+, Sample 1–4) and mice with antigen-induced arthritis (AIA) (AIA 200+, Sample 5–9), and TER119^–^CD31^–^CD45^–^CD51^+^CD200^–^CD105^+^ cells from mice with AIA (AIA 105+, Sample 11–14) performed using (**A**) principal component (PC) analysis, (**B**) hierarchical clustering and (**C**) correlation analysis (Pearson correlation coefficient: white=1, red=0).

Sample similarity analyses were performed using hierarchical clustering, principal component analysis (PCA), and correlation analysis. PCA and correlation analysis reveal clustering of CD105+ cells from mice with AIA (Fig. 1A, C). Hierarchical clustering also separates 4 samples from CD105+ cells from CD200+ cells, but one sample clusters with CD200+ samples (Fig. 1B). In all three analyses, samples of CD200+ cells from mice with arthritis and non-immunized (NI) mice group together (Fig. 1A, B, C).

**Fig. 2:**
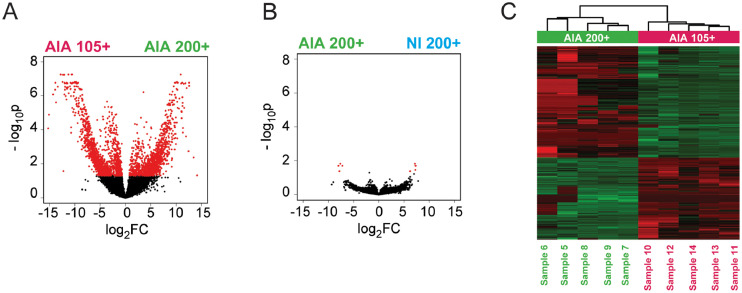
**Analysis of differentially expressed genes.** Differentially expressed genes between sorted synovial TER119^–^CD31^–^CD45^–^CD51^+^CD200^+^CD105^–^ cells from mice with antigen-induced arthritis (AIA) (AIA 200+, Sample 5–9) and TER119^–^CD31^–^CD45^–^CD51^+^CD200^–^CD105^+^ cells from mice with AIA (AIA 105+, Sample 11–14), as well as TER119^–^CD31^–^CD45^–^CD51^+^CD200^+^CD105^–^ cells from non-immunized (NI) mice (NI 200+, Sample 1–4) and mice with AIA were detected using *limma voom*[13]. (**A, B**) Results for comparisons shown on volcano plots, where the negative logarithm value of Benjamini-Hochberg adjusted p value (-log_10_p) for each gene (black dots) is shown in relation to logarithm of fold change (log_2_FC). Differentially expressed genes are shown as red dots (absolute FC >1.5, p (Benjamini-Hochberg) <0,05). (**C**) Differentially expressed genes between AIA 200+ (green) and AIA 105+ (pink) shown with heatmap, where red color represents higher and green color lower gene expression.

Analysis of differentially expressed genes (DEG) detected 2883 genes differentially expressed between CD200+ and CD105+ cells of mice with AIA. Of those, 1227 were upregulated in CD200+ cells and 1656 were upregulated in CD105+ cells (absolute FC >1.5, p (Benjamini-Hochberg) <0,05, Table 3 available at Mendeley Data [11], Fig. 2A, C). Similarity of CD200+ cells from AIA and NI mice is further confirmed by analysis of differentially expressed genes (DEG), where only 9 genes are differentially expressed between those populations (5 upregulated and 4 downregulated, absolute fold change(FC) >1.5, p (Benjamini-Hochberg) <0,05, Fig. 2B, Table 1 available at Mendeley Data [11]).

**Fig. 3:**
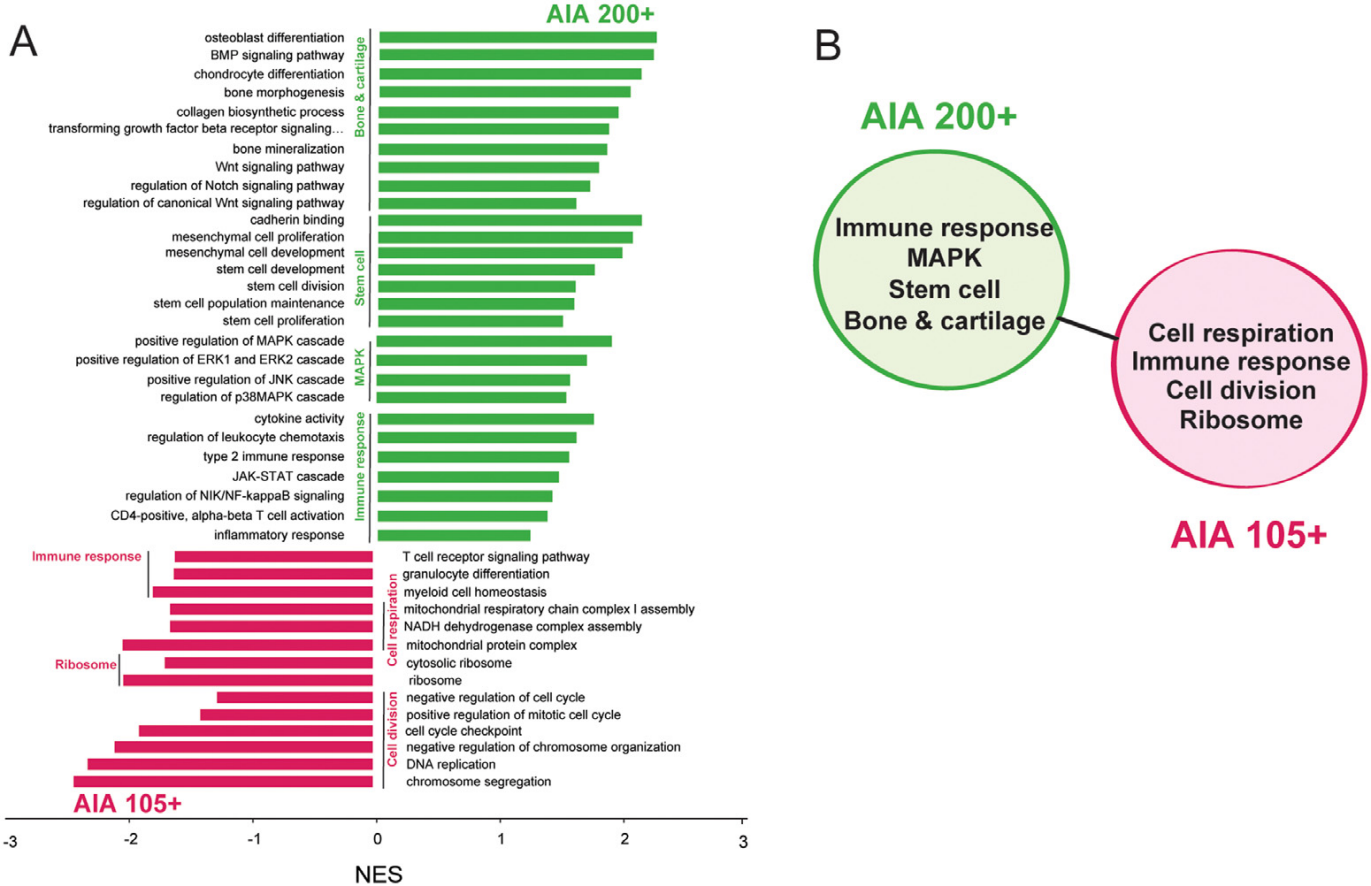
**Gene set enrichment analysis (GSEA).** GSEA analysis for comparison of sorted synovial TER119^–^CD31^–^CD45^–^CD51^+^CD200^+^CD105^–^ cells from mice with antigen-induced arthritis (AIA) (AIA 200+) and TER119^–^CD31^–^CD45^–^CD51^+^CD200^–^CD105^+^ cells from mice with AIA (AIA 105+) was performed on publicly available GO gene sets from biological processes (BP) cell component (CC) and molecular function (MF) categories. Gene sets with Benjamini-Hochberg adjusted p value <0.05 were considered significantly enriched. (**A**) Figure shows normalized enrichment score (NES) for selected representative significantly enriched gene sets in comparisons of AIA 200+ and AIA 105+. (**B**) Functionally similar groups of gene sets enriched in CD200+ AIA in comparison to AIA 105+.

Gene set enrichment analysis (GSEA) showed that CD200+ cells from AIA mice show an enriched expression of genes involved in differentiation of bone and cartilage tissues, Wnt, BMP, TGF-β, Notch, MAPK signaling pathways, and several immune processes, like regulation of differentiation and chemotaxis of leukocytes and chemokine signaling, when compared to CD105+ cells from mice with AIA. In addition, CD200+ cells from AIA mice also show enrichment of genes involved in proliferation and maintenance of stem cells (GSEA, p (Benjamini-Hochberg) <0.05, Fig. 3A, B, Table 3 available at Mendeley Data [12]). On the other hand, CD105+ cells show enrichment of genes involved in cell cycle regulation, DNA replication, cellular respiration, ribosomal processes, and regulation of immune system (GSEA, p (Benjamini-Hochberg) <0.05, Fig. 3A, B).

## Experimental Design, Materials and Methods

### Mice

Eight to twelve-week old female C57BL/6 J mice were used in all experiments. Colonies were maintained at the animal facility of the Croatian Institute for Brain Research, University of Zagreb, School of Medicine, under standard conditions (10 h light and 14 h dark daily, standard chow (4RR21/25; Mucedola, Italy) and water ad libitum). All animal protocols were approved by the Ethics Committee of the University of Zagreb, School of Medicine (380–59–10,106–15–168/235) and the National ethics committee (EP 07–2/2015), and conducted in accordance with accepted standards of ethical care and use of laboratory animals.

### Antigen-induced arthritis (AIA)

AIA was induced as previously described [1]. Mice were immunized by two injections of methylated bovine serum albumin (mBSA, Sigma-Aldrich, St Louis, MO, USA) in complete Freund's adjuvant (CFA, Sigma-Aldrich). Three weeks after the first immunization, arthritis was induced by intra-articular (i.a.) injection of mBSA dissolved in phosphate buffered saline (PBS) into both knees. NI mice were injected with PBS at all timepoints. Injections were performed under tribromoethanol (Sigma-Aldrich) anesthesia. Mice were sacrificed on day 10 after i.a. injection (31 days after primary immunization) by cervical dislocation under tribromoethanol anesthesia, when knees were harvested for synovial cell suspension preparation for fluorescence activated cell sorting (FACS).

### Cell sorting

Knee joints were isolated by cutting tibias and femurs at the level of growth plates. After flushing away any remaining periarticular bone marrow, joints were injected with 1 mg/ml collagenase type IV (Sigma-Aldrich) and incubated for 1 h at 37 °C. Single cell suspensions were prepared by passing the cells through a 100 μm-cell strainer. Non-specific antibody binding was blocked by anti-mouse CD16/CD32 (eBioscience, Thermo Fisher Scientific, 93) for 5 min at RT, and cells were labeled with the following antibodies: CD90.2- fluorescein isothiocyanate (FITC, 30-H12; eBioscience, Thermo Fisher Scientific), CD200-phycoerythrin (PE, OX90; eBioscience, Thermo Fisher Scientific), CD105- phycoerythrin cyanine 7 (PECy7, MJ7/18; eBioscience, Thermo Fisher Scientific), CD45-allophycocyanin (APC; 30-F11; eBioscience, Thermo Fisher Scientific), CD31-APC (390; eBioscience, Thermo Fisher Scientific), TER119-APC (TER-119; eBioscience, Thermo Fisher Scientific), CD51-biotin (RMV-7; BioLegend, San Diego, CA, USA) and streptavidin-APCeFluor780 (eBioscience, Thermo Fisher Scientific). Dead cells were excluded by binding of 4′,6-diamidino-2-phenylindole dihydrochloride (DAPI, Sigma-Aldrich). Cell sorting was performed by a BD FACSAria IIu (BD Biosciences, Franklin Lakes, New Jersey, USA) instrument, as described previously [1]. For each sample, 200–500 cells from selected populations (TER119^–^CD31^–^CD45^–^CD51^+^CD200^+^CD105^–^ or TER119^–^CD31^–^CD45^–^CD51^+^CD200^–^CD105^+^) were sorted directly into cell lysis buffer from Smartseq v4 Ultra® Low Input RNA Kit for Sequencing (TakaRa, Kyoto, Japan) according to the manufacturer's instructions. Sorting parameters were optimized for high purity sorting.

### Library preparation and sequencing

Smartseq v4 Ultra® Low Input RNA Kit for Sequencing (TakaRa) was used to reversely transcribe RNA from lysed sorted cells to first strand cDNA and amplify it. ERCC RNA Spike-In Mix (Invitrogen, Thermo Fisher Scientific, Waltham, MA, USA) was added to lysed cell samples before reverse transcription (RT), so that the final ERCC dilution in RT reaction was 1:65.000 and the final dilution in the PCR mix was 1:100.000. Amplicons were purified using Agencourt AMPure XP Kit (Beckman Coulter, Pasadena, CA, USA). Quality of cDNA amplicons was assessed using High sensitivity cDNA chip and Bioanalyzer instrument (Agilent Technologies, Inc., Santa Clara, CA, USA). One (1) ng of each cDNA amplicon was used for the library preparation using Nextera XT DNA Library Preparation Kit (Illumina, San Diego, CA, USA). Quality and concentration of libraries were assessed using High sensitivity cDNA chip and Bioanalyzer instrument (Agilent) and Qubit™ 1X dsDNA HS Assay Kit and Qubit 2.0 Fluorometer (Invitrogen). Libraries were pooled and approximately 50 million paired end 75 bp reads per sample were sequenced on NextSeq 500 instrument (Illumina) using High Output Kit v2.5 (150 Cycles) (Illumina).

### Bioinformatic analysis

After demultiplexing, the quality of FASTQ files was assessed using FASTQC (https://www.bioinformatics.babraham.ac.uk/projects/fastqc/). Reads were then trimmed with cutadapt [3] to remove Illumina and Smartseq adapter sequences (CTGTCTCTTATACACATCT and AAGCAGTGGTATCAACGCAGAGT). Sequence alignment was done using HISAT2 [4] with default parameters. Samtools [5] was used to create, index and merge BAM files of reads from different lanes belonging to individual samples. In addition to HISAT2 alignment summary, alignment quality was assessed using RSeQC [14] and picard (http://broadinstitute.github.io/picard/). Transcripts were assembled and quantified using StringTie [6], after which analysis was performed in R using Bioconductor packages. The egdeR package [7] was used to normalize count matrices with trimmed mean of M values normalization (TMM) [15]. Genes with sum of counts in all samples lower than 50 and those without Ensembl annotation were removed. The limma voom algorithm [13] from limma package [8] was used to assess the differential gene expression. Genes with absolute fold change (FC) higher than 1.5 and adjusted p value (Benjamini-Hochberg correction) lower than 0.05 were considered significantly changed. GSEA analysis was conducted by ClusterProfiler package using gseGO function [9]. For GSEA analysis, genes were preranked according to signed logarithm (log_10_) of Benjamini-Hochberg adjusted p value, with positive or negative sign given to genes with positive or negative fold change, respectively. Gene sets with p value (Benjamini-Hochberg correction) lower than 0.05 were considered significantly enriched.

## Ethics Statement

All experiments comply with the ARRIVE guidelines and were carried out in accordance with the U.K. Animals (Scientific Procedures) Act, 1986 and associated guidelines, EU Directive 2010/63/EU for animal experiments, or the National Institutes of Health guide for the care and use of Laboratory animals (NIH Publications No. 8023, revised 1978). All animal protocols were approved by the Ethics Committee of the University of Zagreb, School of Medicine (380–59–10,106–15–168/235) and the National Ethics Committee (EP 07–2/2015)

## Declaration of Competing Interest

The authors declare that they have no known competing financial interests or personal relationships, which have, or could be perceived to have influenced the work reported in this article.
